# Critical Care Admission of an HIV Patient with Diabetic Ketoacidosis Secondary to Pembrolizumab

**DOI:** 10.1155/2020/8671530

**Published:** 2020-03-25

**Authors:** John A. Cuenca, Andres Laserna, María P. Reyes, Joseph L. Nates, Gregory H. Botz

**Affiliations:** ^1^Department of Critical Care & Respiratory Care, Division of Anesthesiology, Critical Care and Pain Medicine, The University of Texas MD Anderson Cancer Center, Houston, TX, USA; ^2^Department of Anesthesiology and Perioperative Medicine, The University of Rochester, Rochester, New York, NY, USA

## Abstract

**Background:**

Pembrolizumab is a checkpoint inhibitor that targets the programmed cell death-1 receptor (PD-1) and has shown to be effective against several malignancies, including lung cancer. However, life-threatening immune-related adverse events can result from these immunotherapy treatments. *Case presentation*. A 62-year-old man with HIV, metastatic adenocarcinoma of the lung, and no previous history of diabetes presented to the emergency department with new-onset nausea, vomiting, and generalized weakness. Glucose was 1191 mg/dl, hemoglobin A1c 11%, and potassium 6.9 mEq/L. He had metabolic acidosis with a lactate of 6.6 mmol/L and anion gap of 38 mEq/L, and ketones were detected on the urinalysis. Severe diabetic ketoacidosis was diagnosed, and the patient was admitted to the intensive care unit. Additional investigations showed low C-peptide and negative anti-glutamic acid decarboxylase antibody, anti-insulin antibody, and anti-islet-antigen 2Ab antibody. After ruling out other possible etiologies, pembrolizumab was considered to be the cause of the diabetes and ketoacidosis.

**Conclusions:**

Life-threatening adverse drug events associated with checkpoint inhibitors such as pembrolizumab are on the rise. We recommend to closely follow and monitor patients receiving these immunotherapies. This strategy could lead to early detection and prevention, as well as reduction of more serious life-threatening complications requiring intensive care.

## 1. Introduction

Many types of neoplastic cells avoid the scrutiny of the immune system through several mechanisms. One of the most studied is the overexpression of immune checkpoint proteins that are involved in the maintenance of the peripheral tolerance to self-molecules [1]. The immune checkpoint inhibitors are monoclonal antibodies that improve the ability of the immunologic system to recognize and attack tumor cells. The cytotoxic T cell and dendritic cell response against tumor cells are enhanced through the inhibition of the programmed cell death protein 1 (PD-1), programmed cell death 1 ligand (PDL-1), and cytotoxic T lymphocyte-associated antigen 4 (CTLA-4) [1]. In recent years, checkpoint inhibitors have become standard care for the treatment of several malignancies. For example, pembrolizumab targets the PD-1 protein and has been associated with better overall survival in the treatment of metastatic melanoma and advanced non-small-cell lung cancer [2, 3]. However, by decreasing immune self-tolerance, checkpoint inhibitors can enhance immune cell infiltration into normal tissues and lead to life-threatening immune-related adverse events (IR-AEs) [4]. One of the most common IR-AEs is autoimmune endocrine diseases. Insulin-dependent diabetes mellitus (IDD) has been reported as an endocrine autoimmune toxicity in patients on pembrolizumab [5]. We present a case of diabetic ketoacidosis (DKA) secondary to pembrolizumab in a patient with HIV that required intensive care management.

## 2. Case Presentation

A 62-year-old African-American man with a history of HIV controlled with dolutegravir, emtricitabine, and tenofovir (CD4 600 cells/mm^3^ and an undetectable viral load) and a metastatic adenocarcinoma of the lung (PD-L1 80% and KRAS G12V positive) presented to the emergency department with new-onset nausea, vomiting, progressive fatigue, and generalized weakness. Two weeks before, he had received the third cycle of carboplatin, pemetrexed, and pembrolizumab. At that time, he was hemodynamically stable, tachycardic, nonfebrile, and alert. His blood glucose was 1191 mg/dl, hemoglobin A1c 11% (previously 5.3% before the first dose of pembrolizumab), serum potassium 6.9 mEq/L, 23.2 K/ul leukocytes, BUN 48 mg/dl, creatinine 2.45 mg/dl (baseline 1.12 mg/dl), and lipase levels were 17U/L. He had a metabolic acidosis with pH 7.19, PaCO_2_ 24 mmHg, HCO_3_ 9 mmol/L, lactic acid 6.6 mmol/L, and anion gap 38 mEq/L, and ketones were detected on the urinalysis. The patient was admitted to the intensive care unit with presumed severe DKA of unclear etiology, possibly exacerbated by sepsis. Normal saline (NaCl 0.9%) IV hydration at a rate of 20 mL/kg/hour, 0.15 unit/kg IV bolus of insulin, followed by continuous intravenous insulin infusion at 0.1 unit/kg/hour, bicarbonate infusion, and broad-spectrum antibiotic coverage were initiated following the institutional protocol for DKA. The patient was pan-cultured for bacteria, fungi, and viruses; however, all were negative, and infection was ruled out.

A complete type 1 diabetes antibodies assay was performed. The anti-glutamic acid decarboxylase antibody (anti-GAD), anti-insulin antibody, and anti-islet-antigen 2Ab antibody were negative. C-peptide was 0.2 ng/mL (normal range: 0.5 to 2.0 ng/mL) and immunotherapy-related type 1 diabetes mellitus (T1DM) was diagnosed. The differential diagnoses were explored to find a probable etiology. Highly active antiretroviral therapy could cause hyperglycemia, but the patient had been on antiretroviral therapy for four years and had never presented with hyperglycemia. In the setting of normal serum lipase, a normal pancreatic structure in ultrasound and CT scan, and no signs of exocrine dysfunction, pancreatic insufficiency was also ruled out. In the case of new-onset T2DM, it is highly unlikely for a patient to have such rapidly progressive disease in three months. In addition, the significant rise in hemoglobin A1c after the initiation of the immunotherapy suggested a temporal association between the pembrolizumab and the development of DKA. Having ruled out other causes, new-onset IDD secondary to pembrolizumab was considered the final diagnosis.

After four days in the intensive care unit, there was a marked clinical improvement with resolution of the ketoacidosis and anion gap and control of the blood glucose. The patient was transferred to the ward and was discharged home with a subcutaneous insulin regimen. The immunotherapy was restarted after assessing the adequacy of and the compliance with the insulin regimen. He completed the fourth cycle of carboplatin, pemetrexed, and pembrolizumab and was later transitioned to the maintenance phase with pemetrexed and pembrolizumab. The endocrinology service recommended the administration of intramuscular dexamethasone as prophylaxis for an IR-AE on days 2–4 of each cycle.

## 3. Discussion

Diabetic ketoacidosis is a common presentation of insulin-dependent diabetes mellitus, which may be exacerbated by infection. Our patient was initially assessed for DKA in the setting of sepsis. After finding negative cultures and ruling out other common etiologies, it was concluded that the DKA was caused by an IR-AE from pembrolizumab. This has been previously described in the literature with an estimated incidence of <1% [6].

Immunotherapy-induced IDD has been classified as new-onset T1DM or worsening of preexisting T2DM. Approximately 67% of the IDD cases developed DKA as the first manifestation [5]. There are several hypotheses for the appearance of these IR-AEs. Animal studies have shown that PD-L1 expression in pancreatic beta-cells inhibits the release of cytokines from CD4+ T lymphocytes, thereby preventing the accumulation of CD8+ T lymphocyte in the pancreas [7]. Additionally, when PD-1 and PD-L1 were inhibited in diabetic mice, there was an increase in the number of self-reactive T cells in pancreatic lymph nodes [8]. Thus, the mechanism that underlies the development of immune checkpoint inhibitor-induced IDD is most likely T cell-mediated destruction of pancreatic beta-cells [5]. Interestingly, IR-AEs seem to be related to better tumor response to checkpoint inhibitors therapy [9].

A recent study evaluated nivolumab and pembrolizumab (PD-1) as an emergent cause of diabetes mellitus and found an overall incidence of new-onset IDD of 1.8%, which most frequently occurred after four cycles of the therapy. Out of the 774 patients on pembrolizumab, 11 (1.4%) had T1DM, and 6 (0.8%) had worsening of preexisting T2DM [5]. Despite unsuccessful attempts to use glucocorticoids to reverse the IDD [10], only one case has been reported with spontaneous resolution [5]. As seen in our patient, most subjects with checkpoint inhibitor-induced IDD remain permanently diabetic.

In our patient, the presentation of IR-AEs occurred in the context of an HIV infection. Until recently, most clinical trials have excluded patients with HIV requiring immunosuppressive treatment to avoid the potential risk of exacerbating the viral infection [11]. HIV infection poses an increased risk of autoimmunity [12], usually manifest as systemic lupus erythematosus, rheumatoid arthritis, Grave's disease while the immune system is still competent, or as Reiter's syndrome, psoriasis, and diffuse infiltrative lymphocytosis syndrome during the AIDS phase [13]. For this reason, it could be hypothesized that HIV patients are at increased risk to develop IR-AEs. However, HIV infection does not increase the risk of diabetes [14]. A small multicenter phase 1 clinical trial did not find evidence of IDD within an HIV population treated with pembrolizumab [15]. There was a reported case of an HIV patient who received nivolumab for Hodgkin's lymphoma and developed anti-GAD 65 positive IDD [16]. Our patient did not have detectable anti-GAD 65. These antibodies are present in up to 85% of patients with T1DM. Both patients were on highly active antiretroviral therapy, received additional cytotoxic chemotherapy, and exhibited an adequate tumor response to PD-1 inhibitors.

Due to the rise of non-AIDS defining malignancies among patients with HIV [17] and since the use of PD-1 inhibitors will continue to rise, it is essential to evaluate a possible increased risk of IR-AEs in HIV patients. Future clinical studies should assess this association.

Life-threatening adverse drug events associated with the use of checkpoint inhibitors such as pembrolizumab are becoming more prevalent. By reporting this association, we aim to raise clinicians' awareness of the vast array of these reactions. We also recommend a thorough follow-up and monitoring of patients at high risk of developing IR-AEs. Disease-specific screening should be investigated in large prospective studies. This strategy could lead to early detection and prevention, as well as reduction of more serious life-threatening complications requiring intensive care. Failing to prevent the already known complications would reduce the overall quality of care and could lead to death.
